# Toxicity of the Diatom Genus *Pseudo-nitzschia* (Bacillariophyceae): Insights from Toxicity Tests and Genetic Screening in the Northern Adriatic Sea

**DOI:** 10.3390/toxins14010060

**Published:** 2022-01-15

**Authors:** Timotej Turk Dermastia, Sonia Dall’Ara, Jožica Dolenc, Patricija Mozetič

**Affiliations:** 1 Marine Biology Station Piran, National Institute of Biology, 6330 Piran, Slovenia; patricija.mozetic@nib.si; 2 International Postgraduate School Jožef Stefan, 1000 Ljubljana, Slovenia; 3 National Reference Laboratory for Marine Biotoxins, Centro Ricerche Marine, 47042 Cesenatico, Italy; sonia.dallara@centroricerchemarine.it; 4 Institute of Food Safety, Feed and Environment, Veterinary Faculty, University of Ljubljana, 1000 Ljubljana, Slovenia; jozica.dolenc@vf.uni-lj.si

**Keywords:** Adriatic, *dabA*, domoic acid, *Pseudo-nitzschia galaxiae*, ITS

## Abstract

Diatoms of the genus *Pseudo-nitzschia* H.Peragallo are known to produce domoic acid (DA), a toxin involved in amnesic shellfish poisoning (ASP). Strains of the same species are often classified as both toxic and nontoxic, and it is largely unknown whether this difference is also genetic. In the Northern Adriatic Sea, there are virtually no cases of ASP, but DA occasionally occurs in shellfish samples. So far, three species—*P. delicatissima* (Cleve) Heiden*, P. multistriata* (H. Takano) H. Takano, and *P. calliantha* Lundholm, Moestrup, & Hasle—have been identified as producers of DA in the Adriatic Sea. By means of enzme-linked immunosorbent assay (ELISA), high-performance liquid chromatography with UV and visible spectrum detection (HPLC-UV/VIS), and liquid chromatography with tandem mass spectrometry (LC-MS/MS), we reconfirmed the presence of DA in *P. multistriata* and *P. delicatissima* and detect for the first time in the Adriatic Sea DA in *P. galaxiae* Lundholm, & Moestrup. Furthermore, we attempted to answer the question of the distribution of DA production among *Pseudo-nitzschia* species and strains by sequencing the internal transcribed spacer (ITS) phylogenetic marker and the *dabA* DA biosynthesis gene and coupling this with toxicity data. Results show that all subclades of the *Pseudo-nitzschia* genus contain toxic species and that toxicity appears to be strain dependent, often with geographic partitioning. Amplification of *dabA* was successful only in toxic strains of *P. multistriata* and the presence of the genetic architecture for DA production in non-toxic strains was thus not confirmed.

## 1. Introduction

*Pseudo-nitzschia* H. Peragallo is a genus consisting of 54 confirmed species of diatoms, about half of which have been confirmed as producers of the neurotoxin domoic acid (DA) [1,2]. There are several methods for detecting DA that have evolved over time. Shellfish-monitoring programs use the standard reference method—liquid chromatography with ultraviolet detection, which is sufficient because threshold concentrations are usually high (20 µg/kg shellfish tissue). Immunoassays are also readily available from commercial manufacturers and offer high sensitivity and throughput. Finally, liquid chromatography coupled with tandem mass spectrometry (LC-MS/MS) is the main method used in research today, as it offers high throughput and analytical precision. Several mechanisms for the induction and upregulation of domoic acid have been proposed, often with conflicting evidence. From the synthesis of factors affecting the production of DA presented in [3], it appears that the physiological state of the cell has a significant influence although the evolutionary purpose of the production of DA is not fully understood. Originally, it was proposed that DA is a chelating agent for iron and copper ions [4,5,6] although there is still conflicting evidence for this hypothesis [7,8]. Nonetheless, the understanding of toxin regulation and physiology has improved significantly recently, particularly with the discovery of the DA biosynthetic pathway, where four enzymes (DabA–D) coded by four genes (*dabA*–*D*) were discovered [9]. Furthermore, it was established that DA production is induced by copepod grazer cues [10,11] although an increase in DA concentration did not significantly affect grazing in these studies. Recently, however, new evidence has been presented for the deterrent function of DA against grazers [12]. Whatever evolutionary or ecological advantage the production of DA may provide, questions remain as to why some strains of the same species produce it and others do not and how the ability to produce DA is distributed along the phylogenetic tree of *Pseudo-nitzschia*. This is particularly interesting since some studies suggest that species that do not produce DA do not benefit from the addition of DA into their iron-limited growth media [8]. Conversely, the deterrent effect on grazers seems to be of great benefit to DA-producing strains, so it could be assumed that the loss of this ability would be detrimental to such strains. The discovery of nzyme-encoding genes involved in the biosynthetic pathway of DA production [9] provides an opportunity to trace gene distribution and structure within the genus *Pseudo-nitzschia* and beyond and to answer these questions.

The aim of this work was to determine the toxicity potential of several *Pseudo-nitzschia* species found in the Gulf of Trieste (GoT), the northern Adriatic [13], and to complement these results with molecular data to determine whether the production of DA is phylogenetically linked. In this context, we also investigated whether the *dabA* gene is present in strains that we found did not produce DA. The GoT is a nutrient-rich environment with occasional phosphorus limitation. The temperature rarely drops below 7 °C in winter and can exceed 28 °C at the surface in summer. Although potentially toxic *Pseudo-nitzschia* species occasionally bloom here [13,14], DA is rarely found in shellfish and is generally not harmful to the food industry [15]. The diversity of *Pseudo-nitzschia* in the Gulf of Trieste has only recently been elucidated [13], while numerous reports are available for other regions of the Adriatic Sea, e.g., [16,17,18,19,20]. Species richness is comparable to that of other coastal regions of the Mediterranean and other temperate zones, while seasonality and species occurrence seem to be localized to some extent. Reports on toxicity are much sparser, but the presence of DA in cultures of *P. delicatissima* [17], *P. multistriata* [21], and recently *P. calliantha* [22] has been reported. The latter was previously suspected based on circumstantial evidence derived from the analysis of toxin-positive mussel samples and the accompanying net trawls in which *P. calliantha* and *P. pseudodelicatissima* (Hasle) Hasle cells were found [19,23].

We tested six species using different methods and report toxin production in three species, namely *P. multistriata, P. delicatissima*, and—for the first time in this area—in *P. galaxiae*. Several strains of each species were studied, and three morphological types were also recognized in *P. galaxiae* [13,24]. We complement our results with a global perspective on the phylogeny of toxic and non-toxic *Pseudo-nitzschia* and as well provide preliminary insight into the distribution of the *dabA* gene.

## 2. Results

### 2.1. Toxicity of Individual Strains

Thirty-three strains belonging to six species of *Pseudo-nitzschia* were analyzed for the content of DA. Table 1 shows the toxicity results for each strain tested. The method with the lowest limit of detection (LOD) was the ELISA method, with a LOD of 0.17 ng/mL of DA and a limit of quantification (LOQ) of 0.5 ng/mL. HPLC-UV had a LOQ of 2 µg∙mL^−1^, and LC-MS/MS had a LOQ of 0.8 µg∙mL^−1^. As you can see, some strains were confirmed to be toxic only by the ELISA test, while they did not prove positive in HPLC-UV and in the LC-MC/MS method. This prompted us to retest these samples with ELISA at lower dilutions to confirm the presence or absence of DA. Most retests resulted in concentrations below LOQ, with the exception of *P. galaxiae* strain B3S, where the concentration was still above the limit of quantification. There were also many borderline strains that had concentrations below 0.5 ng/mL (LOQ), but we could not rule out their toxicity because the absorbance was lower than the negative standard values, indicating some competitive binding in the ELISA. *P. galaxiae* strains BAT2 and B2S also showed inconclusive results, as the dissolved DA (dDA) fraction gave higher measured concentrations than the total DA (tDA) fraction. On repeated analysis, both particulate (pDA) and dDA in B2S were below LOQ, while in BAT2, the concentration of pDA was higher than dDA although we could not quantify it again as the signal remained above quantification. We could not confirm toxin production in strains of *P. mannii* Amato & Montresor and *P. subfraudulenta* (Hasle) Hasle and *P. calliantha* although for one strain of *P. calliantha* and one strain of *P. mannii*, results indicated minute concentrations below the LOQ of the ELISA assay.

### 2.2. DA Production in Different Growth Phases

**Figure 1:**
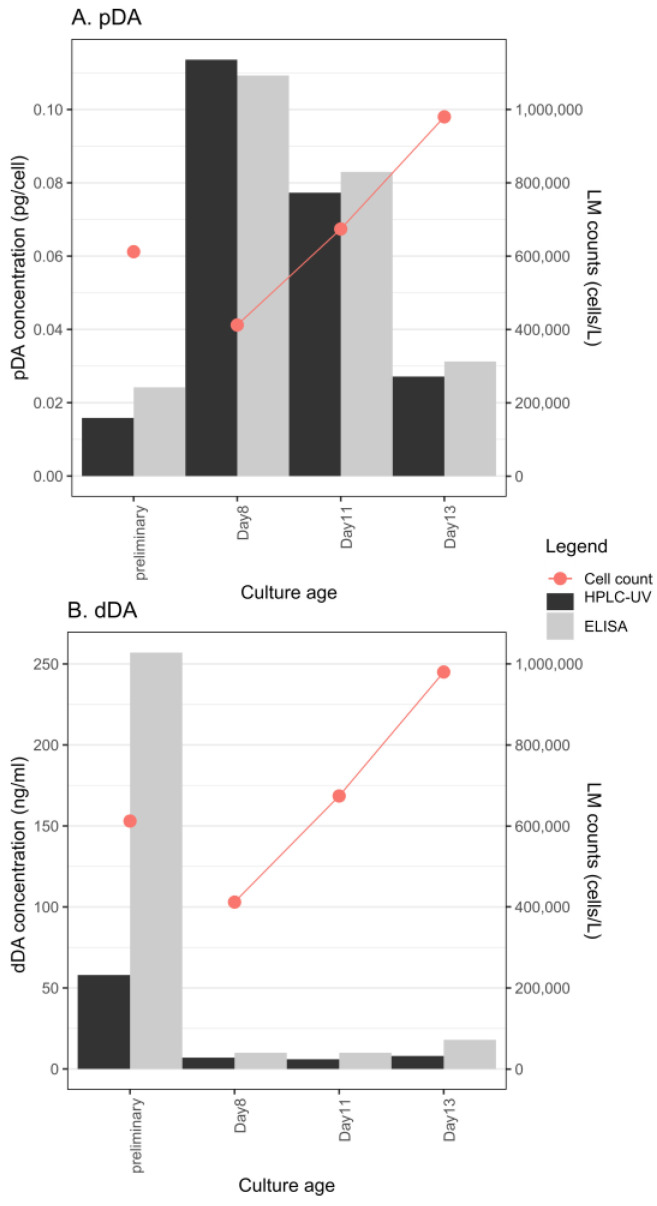
(**A**) Change in pDA content in *P. multistriata* strain 119-A4; (**B**) change in dDA content in P. multistriata strain 119-A4. The preliminary measurements were part of the screening experiment presented in Table 1.

We observed a decreasing trend in particulate toxin content with increasing cell density in *P. multistriata* strain 119-A4 (Figure 1A), while dDA increased slightly only on the last day of measurements (Figure 1B). It can be seen that the initial screening yielded a similar cell number as on day 11 but a completely different concentration for both the pDA and dDA fractions. While the pDA fraction was lower in the initial screening compared to day 11, the dDA fraction was significantly higher. In our case, the toxin was already produced in the exponential growth phase, while measurements in the stationary phase were not performed. After the initial confirmation of toxicity in *P. delicatissima* strain 119-B3, we could not confirm DA in the experiment where DA was sampled during different growth stages. The concentration of DA was probably very low in this case since the HPLC-UV method could not detect DA even in the initial screening (Table 1). We see here that the measurements between the HPLC-UV and ELISA methods are comparable and give very similar concentrations, except in the case of the first screening with dDA, where the ELISA method gives much higher concentrations. Reliable concentrations of pDA and dDA for the other strains tested in different growth phases could not be determined and so are not reported here.

### 2.3. Phylogeny and Toxicity

**Figure 2:**
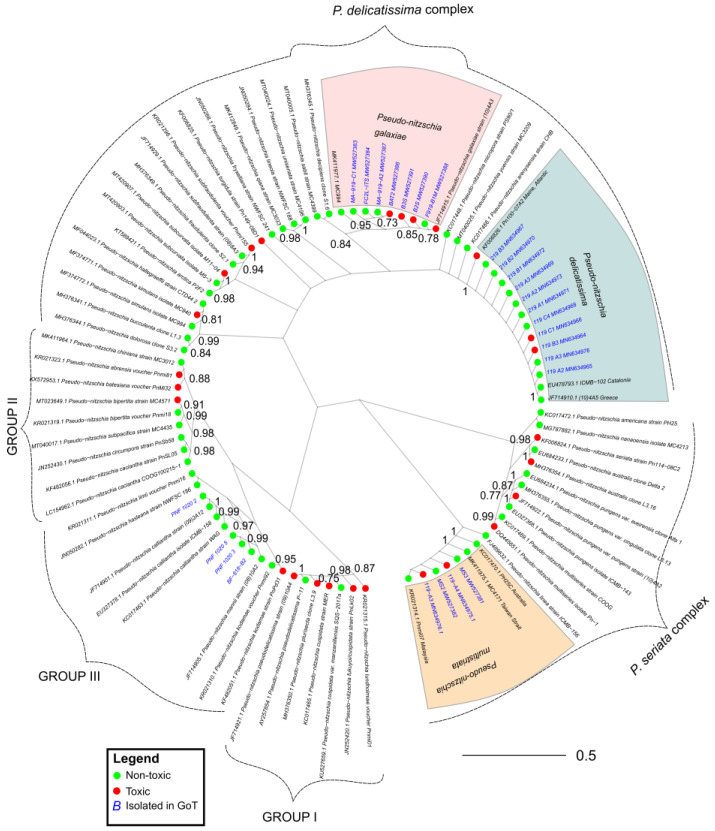
Maximum likelihood (ML) tree of the ITS2 marker constructed using 94 sequences gathered from GenBank (https://www.ncbi.nlm.nih.gov/genbank/; accessed on 19 November 2021), using the TVM + G + I evolutionary model and 10,000 bootstraps of the tree space. Only bootstrap supports higher than 0.7 are shown. >0.95 is considered high support. Note that not all strains presented in Table 1 are included because ITS sequences of some could not be obtained.

**Figure 3:**
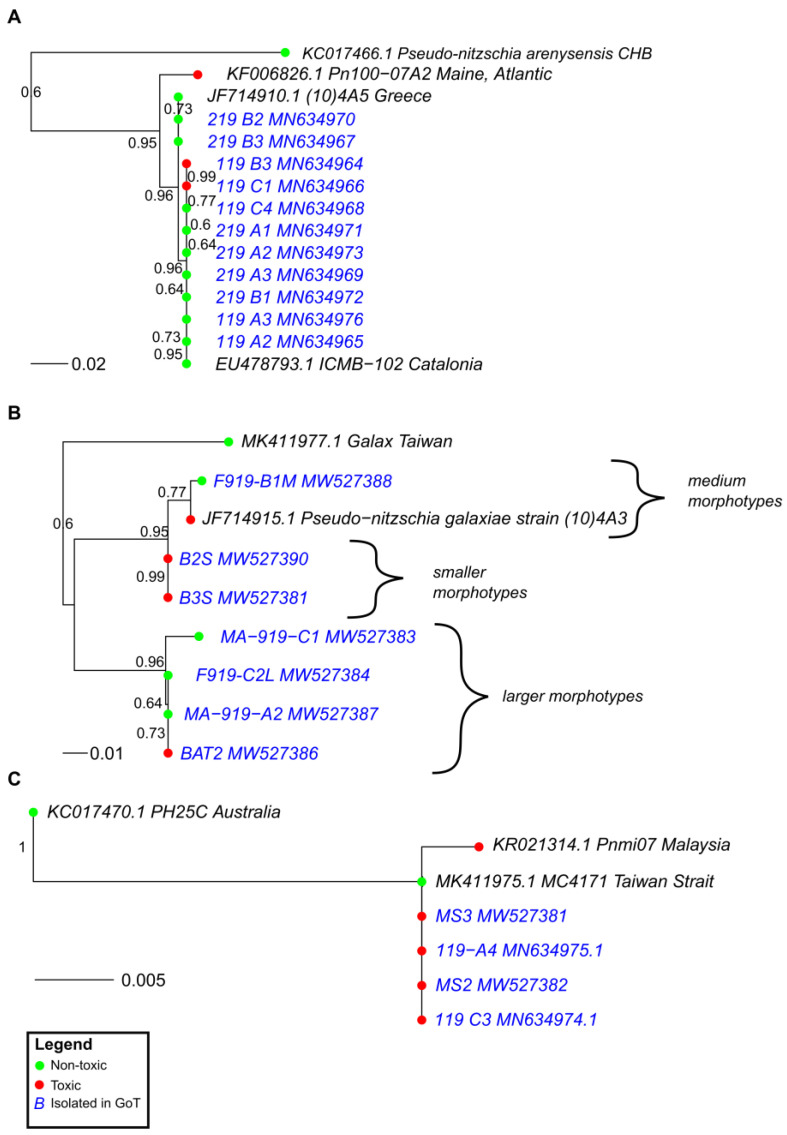
Pruned trees from Figure 2. (**A**) Tree of *P. delicatissima*; (**B**) tree of *P. galaxiae*; (**C**) tree of *P. multistriata*. The trees were drawn using the same conditions as the tree in Figure 2. Bootstrap supports higher than 0.5 are shown.

**Figure 4:**
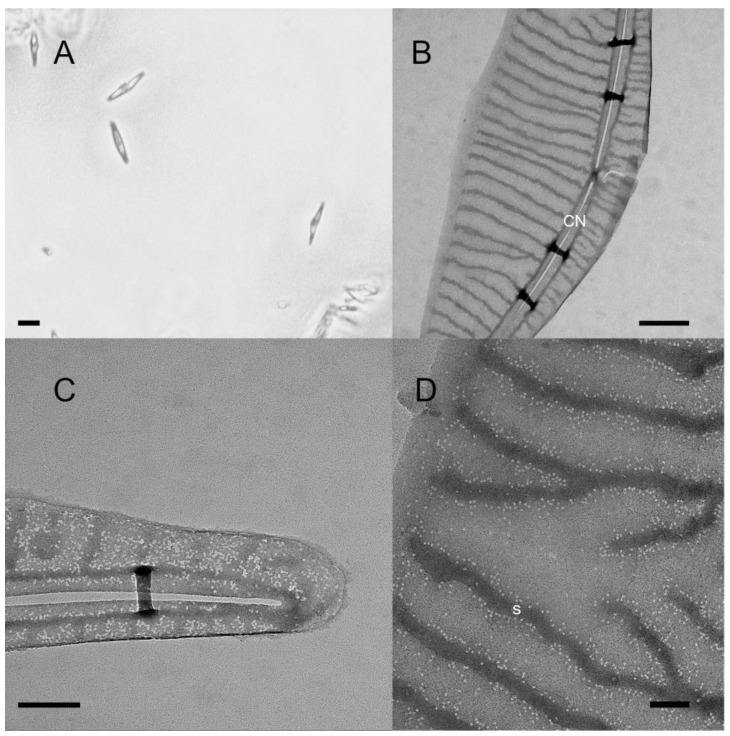
*P. galaxiae* small morphotype, strain B3S. (**A**) LM image of the cells in culture. Scale bar = 5 μm; (**B**) Transmission electron microscope (TEM)image of the cell valve at the position of the central nodule (CN). Scale bar = 500 nm; (**C**) TEM image of the cell tip with the random distribution of poroids. Scale bar = 200 nm; (**D**) TEM image showing the tightly packed poroids along the striae(s) with rare poroids in the interstrial space. Scale bar = 100 nm.

**Figure 5:**
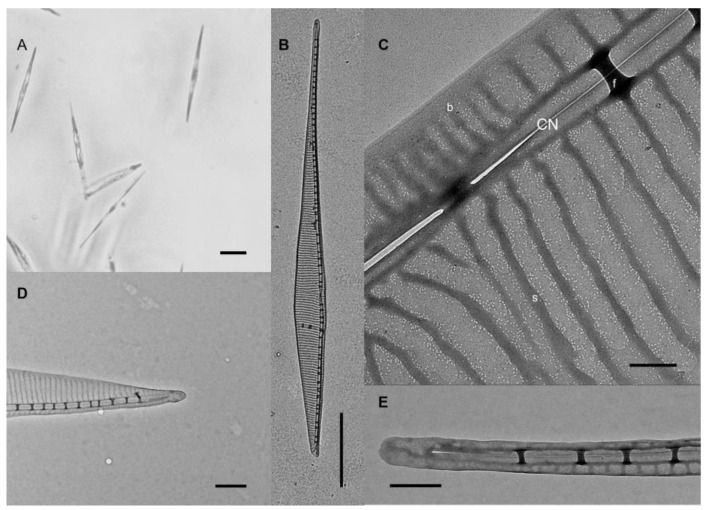
*P. galaxiae* strain BAT2 with a distinct bat-like morphology. Note that not all cells of the culture had this morphology and that the environmental isolate from which the culture was grown demonstrated this morphology. (**A**) LM image of the cells in culture. Scale bar = 10 μm; (**B**) TEM image of the entire valve. Scale bar = 5 μm; (**C**) Detailed TEM image of the valve and the central nodule (CN), with the visible random distribution of several poroids between striae(s), fibulae, and the band (**B**). Scale bar = 200 nm; (**D**) TEM image of cell tip at the unusually shortened end. Scale bar = 1 μm; (**E**) TEM image of the cell tip at the normally elongated end. Scale bar = 500 nm.

Our comprehensive phylogenetic analysis based on the ITS2 sequences of all species of *Pseudo-nitzschia*—where both ITS2 sequence data and toxicity data were available—shows that all major lineages of the genus harbor strains and species that are both toxic and non-toxic (Figure 2). The group with the lowest number of toxic strains was Group III *sensu* [25] although we see that a toxic *P*. kodamae S.T. Teng, H.C. Lim, C.P. Leaw, & P.T. Lim strain is included in this group. We also know that *P. calliantha*, which is a member of Group III, can be toxic, but no ITS sequences of toxic strains were available in GenBank. In some cases, toxic and non-toxic strains were separated by high statistical support. We see this pattern in *P. australis* Franguelli*; P. bipertita* S.T. Teng, H.C. Lim, & C.P. Leaw*; P. galaxiae; P. kodamae*; *P. multiseries* (Hasle) Hasle; *P. subcurvata* (Hasle) Fryxell; *P. subfraudulenta*; and *P. pseudodelicatissima*. In some cases, such as with *P. bipertita*, *P. pseudodelicatissima*, and *P. subfraudulenta*, the differing strains were from different geographical areas, while in other species, strains were from the same area (Table S1). The differences between geographically separated as well as non-separated strains are most pronounced in *P. galaxiae*, which stands out when we take a closer look at the phylogeny (Figure 3B). *P. galaxiae* shows major genetic differences that are consistent with the morphological characterization. Therefore, *P. galaxiae* is hereafter referred to as the *P. galaxiae* species complex although the phylogenetic position of these different strains has not yet been clarified. We see that two major clades have emerged, one consisting mainly of non-toxic larger strains, while the other consisted of well-separated medium and small strains, including three from this study (Figure 4). Both clades harbored a strain that was an exception to this rule although the BAT2 strain was morphologically quite distinct from the other three strains, exhibiting a peculiar baseball bat-like morphology (Figure 5). Strain (10)4A3 from Greece was also a medium-sized strain.

Species that we examined in more detail, since we had many strains available for toxicity testing, were also *P. delicatissima* (Figure 3A) and *P. multistriata* (Figure 3C). We see that in both cases, the genetic divergence of strains on the ITS2 marker was not as large as with the *P. galaxiae* species complex and that identical or slightly-divergent strains appeared to be both toxic and non-toxic. This is particularly evident in *P. multistriata*, which showed very little divergence. In *P. delicatissima*, the divergence was somewhat greater; in particular, the toxic strain PN100-07A2 from the western Atlantic was separated from ours and other Mediterranean strains, two of which were toxic. Unfortunately, we were unable to obtain ITS sequences of the *P. multistriata* strains that were found to be non-toxic in our analysis as well as the non-toxic *P. subfraudulenta* and *P. mannii* strains.

### 2.4. dabA Gene Screening in Toxic and Non-Toxic Strains

PCR with primers published in [9] did not yield specific products with any species, and so, we redesigned primers for the *dabA* gene using the available public sequences, which included a *P. multiseries* sequence from [9] and an incomplete *P. multistriata* sequence from [26], which came from genome sequencing. The designed primer amplified the gene in four of the sequenced *P. multistriata* strains (Table S1). The amplified product was approximately 1450 bp long and contained the intronic region, which was removed from the sequences after alignment with the only two published sequences. The product obtained was a partial gene sequence, as the primers designed in [9] did not yield a specific product, and so, the primers had to be designed internally on the available sequences. The *P. multistriata* sequences obtained were highly conserved and had only a few ambiguous sites, but these were all on degenerate codon positions and did not affect the amino acid sequence. The sequences all aligned well with the published sequence from the *P. multistriata* genome, which was incomplete, because it contained undefined positions, which resulted in a translated amino acid sequence with multiple stop codons. The published sequence of *P. multiseries* (MH202990) is 84% identical to the sequences of our *P. multistriata* strains, whereas the translated sequences show 89% similarity to the DabA protein of *P. multiseries*. There is significant structural similarity identified by homology modelling in Phyre2 (Figure S1), highlighting the functionality of the *P. multistriata* enzyme in producing DA. Our attempt to amplify the *dabA* gene in other species of *Pseudo-nitzschia* using the same primers did not yield specific products. After we purified, isolated, and sequenced the nonspecific bands from the other species, BLAST (https://blast.ncbi.nlm.nih.gov/Blast.cgi; accessed on 6 May 2021) did not yield any highly similar hits. Thus, we confirmed that all toxin-producing strains of *P. multistriata* harbored the *dabA* gene, while we were unable to resolve the dilemma of whether non-toxic strains have the genetic capacity to produce DA or whether they actually lack the required genes. In any case, based on the data we have from two closely related *Pseudo-nitzschia* species—*P. multiseries* and *P. multistriata*—the *dabA* gene is not highly conserved with respect to its nucleotide sequence, and it may well be that the primers we used did not work in *P. galaxiae* and *P. delicatissima* since these species are the furthest from the *P. seriata* (Cleve) H. Peragallo species complex in the phylogenetic tree (Figure 3).

## 3. Discussion

### 3.1. Toxicity

The results of the toxicity analyses complement the studies from the northwestern Adriatic in that mildly toxic strains of *P. delicatissima* and *P. multistriata* are present in the northeastern Adriatic Sea as well. However, the toxicity levels of *P. delicatissima are* 25-times higher (~1.5 fg cell^−1^) than those reported in the northwestern Adriatic (0.063 fg cell^−1^) [17]. During this study, we realized that many factors can affect pDA concentrations, including sample preparation, counting errors, culture condition, and other factors related to the production of DA that we did not control (reviewed in [3,27]). This was particularly evident in the experiment monitoring DA production of *P. multistriata* strain 119-A4 at different phases of growth, where the initial screens differed greatly from the concentration measured during the experiment. Regarding the threat of *P. multistriata* and *P. delicatissima* to the ecosystem and industry in our region, *P. delicatissima* from the northern Adriatic appears to be a mildly toxic species that can reach bloom abundances, especially in spring [13], although shellfish toxin monitoring programs did not detect any DA in shellfish in this period [15,28]. *P. multistriata*, in contrast, appears to have a higher cellular content of DA and also higher than strains from the northwestern Adriatic [21] and the recently identified Peruvian strains [29] but comparable to some strains from the Gulf of Naples [30,31]. However, this species rarely proliferates into high-abundance blooms and has only been detected in the winter months [13].

Furthermore, we can report toxic strains of *P. galaxiae*. These findings are preliminary, as no toxicity could be confirmed by chromatographic methods although repeated ELISA screens confirmed the presence of DA in at least one strain (B3S). DA was found only in small and medium morphotypes of the species [13,24] and even in these only in dDA during the first test, leading to some inconsistencies with pDA calculations. We attributed this to procedural errors and therefore repeated the ELISA with these samples, which resolved this discrepancy somewhat, at least for strain B3S. *P. galaxiae* also showed the greatest genetic divergence between different strains, which was also true for strains isolated on the same day and at the same location (see [13]). These results may additionally suggest that *P. galaxiae*, as now described, is indeed a species complex, which has been suggested previously [13,32] and is also supported by our morphological and toxicological observations. Our data contribute valuable information on the toxicological and phylogenetic position of this species. *P. galaxiae* was so far found to be toxic only in the Aegean Sea (Greece) and even here with trace levels of DA in culture [33]. All other studies investigating *P. galaxiae* toxicity found this species to be non-toxic ([1] and references therein). This is thus the first unequivocal report on *P. galaxiae* toxicity. This species is known to grow to high abundances both in culture—over 1 million cells per mL—and in the environment (unpublished data from Gulf of Trieste). However, due to the small size of the cells, similarly to *P. delicatissima*, it is probably not a threat.

Finally, the possible detection of DA in *P. calliantha*—even if below ELISA quantification—would need further investigation. However, it would not be surprising since DA has only recently been found in Adriatic *P. calliantha* strains [22] although it was previously suspected [19,23]. Conversely, the indication that even some *P. mannii* strains may be toxic is surprising—as this species has not been confirmed to be toxic before [1,34]—although we do not have enough evidence to confirm this.

The onset of production of DA in cultures depends on the species and strains [3] although in most species, DA production starts in the late exponential phase and increases during the stationary phase. We only had one stationary phase sample in our experiment. Our results corroborate those of [35], who found decreasing pDA levels with increasing cell number in *P*. cf. *pseudodelicatissima*, but contrast with the results of [31,36], who found increasing pDA concentrations with increasing cell number in *P. multistriata* and *P. multiseries*, respectively. In the study of [7], the pDA concentration remained constant with increasing abundance of *P. multiseries* although it decreased dramatically when DA was added externally to the growth medium, albeit during increased copper stress. The decrease in pDA concentration with increasing cell concentration may be due to an as-yet unknown quorum-sensing strategy of the cells to accommodate increasing DA in the environment by producing less DA [37]. Quorum-sensing responses could also depend on the resident bacterial community, so differences between strains and cultures would not be unexpected. However, we do not have sufficient data to conclude that this strategy is indeed responsible for our observed results. Our results also suggest that comparing DA concentrations of point samples between studies and basins is not useful because conditions and culture states can vary considerably [3]. 

Finally, we point to some of the inconsistencies in the measurements of pDA in MS2 and MS3, which prompted us to retest these samples with HPLC-UV and LC-MS/MS. However, the LC -MS/MS method used to screen mussel samples had a LOQ of 0.8 µg/mL and so was too high to detect DA in our culture samples with the exception of one strain (PN0DB2131216-C, GenBank 28S accession: MK682491.1), which was analyzed on another occasion. In addition, the repeated tests were performed on samples that had been stored in the original growth medium for six months to over a year after the initial ELISA screens, which may have resulted in some toxin degradation [38,39]. In contrast, the strain positive for DA in LC-MS/MS was specifically prepared for LC-MS/MS analysis and tested shortly after extraction.

### 3.2. Phylogenetic Relationships between Toxic and Non-Toxic Strains

To our knowledge, this is the first attempt to relate the toxicity of *Pseudo-nitzschia* strains to their phylogenetic relationship although speculation that ITS type is not related to the ability to produce DA has been made previously for *P. multistriata* [31]. This has also been shown for yessotoxin-producing dinoflagellates, where no correlations were found between toxicity and phylogenetic position [40]. Our study provides conflicting evidence for this question. Clearly, the ability to produce DA is widespread in the phylogenetic tree of *Pseudo-nitzschia* although it is possible that it was lost several times during the evolutionary history of the genus and its species. The genetic similarity of *P. delicatissima* and *P. multistriata* strains in relation to their toxicity suggests that toxicity in these two cases is irrelevant to the ITS phylogeny and may support the previously discussed idea that the physiological state of the culture determines whether or not DA is produced, where gene expression may also play a role [41].

In contrast, in *P. subfraudulenta*, *P. subcurvata*, *P. australis*, *P. pseudodelicatissima*, *P. multiseries*, and *P. galaxiae*, toxic and non-toxic strains were separated by high bootstrap support that was in certain cases also related to the geographic origin of the strains. This may contribute to the idea that genes required for DA production were lost in relatively recent evolutionary history or that the ability to produce DA is related to the environmental conditions to which strains are acclimatized. Conversely, the differential production of DA in strains from the same area cannot be explained following this logic. In any case, it appears that the ancestral state had the ability to produce DA, which also explains the occurrence of this toxin and its analogs in some other genera of diatoms and even red algae [42,43,44]. This is unless the biosynthetic apparatus was acquired during evolutionary history by horizontal gene transfer mechanisms perhaps on multiple occasions, as was suggested by [45] and which is the presumed pathway for the acquisition of saxitoxin production in dinoflagellates [46]. Horizontal gene transfer in protists is perhaps a neglected phenomenon and may be exacerbated by widespread viral infections [47]. For this hypothesis to be examined, a clear evolutionary role of DA should be established, which is at the moment not the case since proposed roles range from grazer deterrence [10,11,48] to metal chelation [4,5,6], while strains and species are all known to fare well in the absence of DA production. On the other hand, the competitive advantage of DA production may be clearer when local environmental conditions are considered. The third possibility is that the toxin was simply not produced at the time of sampling or that the methods were not sensitive enough, as we have also shown in some cases in this work. To prove this, we would need to trace the genes responsible for the production of DA in the genomes of several species and strains, which is what we attempted next.

### 3.3. DabA in Selected Strains

For the first time since the discovery of the DA biosynthetic pathway [13], we identified the *dabA* gene in a species other than *P. multiseries*. Although the *dabA* sequence of *P. multistriata* was deposited in GenBank (https://www.ncbi.nlm.nih.gov/genbank/, accessed on 14 December 2021) by genome sequencing [26], which allowed us to design primers, we filled in the missing sites in the genome sequence that made it untranslatable. The secondary structure of the protein appears to be conserved between *P. multistriata* and *P. multiseries*, although the nucleotide sequence is only 84% similar, which may contribute to the fact that the primers used did not amplify the gene in other species tested. *P. multistriata* and *P. multiseries* are closely related, whereas *P. calliantha, P. mannii, P. galaxiae*, and *P. delicatissima* are more distantly related ([25], Figure 2). For the strains for which good products were not obtained, the cause could be a missing gene or poor primers. If the latter is the case, this may not be a trivial task since our preliminary data show that the genetic as well as amino acid sequence differences between the closely related *P. multiseries* and *P. multistriata* are quite large. Therefore, further genomic and transcriptomic experiments with other *Pseudo-nitzschia* species—particularly those from Groups I–III and the *P. delicatissima* complex—need to be performed to populate the reference databases, which will facilitate the design of more universal *dabA* primers. 

Recently, a transcriptomic study showed that only *dabA* and *dabD* of the *dabABCD* cluster were expressed in DA-producing *P. pungens*, and only *dabD* was found in *P. fraudulenta* transcripts. In contrast, *P. australis* expressed all four genes [41]. In the future, these transcriptomes could be mined to obtain sequence data and design new primers. However, there is also the possibility that the gene cluster is completely absent from the genome of some species or strains that are not actually toxic [1] although such an explanation is unlikely. This would indicate either multiple deletions or insertions of the gene cluster during the evolutionary history of the genus. Such a mechanism could imply either the horizontal gene transfer, discussed earlier, which is a plausible explanation for the occurrence of DabA analogs in red algae [45], or ongoing hybridization, which has, however, been demonstrated in the genus [48,49].

There is an idea that measuring the copy number of genes involved in the synthesis of DA—e.g., by qPCR—may be the key to accurately predict the threat of ASP. At the moment, however, the design of suitable probes is hampered by the lack of sequences from different species and could perhaps only be developed for *P. multiseries* and now with our data for *P. multistriata*. We acknowledge that there are other genes involved in the biosynthesis of DA [9,11] that we did not examine in this work and that may prove to be more conserved and thus better targets for such efforts. However, the focus of this work was on the toxicity profiles of NE Adriatic strains, and we hope that this work will open new opportunities for the study of gene expression and the discovery of genes involved in the synthesis of DA. 

## 4. Materials and Methods

### 4.1. Species Cultures

The cultures used in toxicity tests were obtained from the National Institute of Biology—Marine Biology Station Piran, Slovenia culture collection and grown in 50 mL of L1 medium in 150-mL Erlenmeyer flasks, as described in [13]. Cultures of *P. calliantha, P. delicatissima, P. galaxiae*, *P. mannii*, *P. multistriata*, and *P*. s*ubfraudulenta* were used for this study.

### 4.2. Domoic Acid in Cultures

All cultures were tested at one point in the stationary growth phase. Fifty μL of the culture were taken in triplicate for counting under an Olympus BX51 microscope (Olympus, Tokyo, Japan) in a Fuchs–Rosenthal chamber (FRC). Then, depending on the number of cells, 10–20 mL of the culture were taken in duplicate. One of the replicates was sonicated on ice at 40 Hz for one minute to break up the cells and release the toxin. This sample was then filtered through a Millex 0.22-µm syringe filter (Merck-Milipore, Darmstadt, Germany) to remove debris, and the filtrate was stored at −20 °C. This represented the total DA (tDA). The other sample was centrifuged at 4500× *g* for 30 min, and the supernatant was stored at −20 °C. This was the dissolved DA (dDA) fraction. Since the centrifugation process is not perfect, the supernatant was recounted in the FRC to account for the remaining live cells. This was done as follows: the top layer of the supernatant was counted in duplicate, then a fraction of the supernatant was pipetted for toxin analysis. Then, the bottom layer of the supernatant was counted in duplicate. The cell count of both layers was then averaged, assuming a gradual increase in cell count from top to bottom in a centrifuged tube. To obtain the particulate DA concentration (pDA—toxin stored in cells), we subtracted the dDA fraction from the tDA and divided the resulting concentration by the number of cells in the lysate. A total of 33 strains from six species were tested.

For three strains of *P. multistriata* (119-A4, MS2, and MS3) and one strain of *P. delicatissima* (119-B3) that tested positive for DA in the first phase, an additional experiment was performed, namely the monitoring of DA at different stages of culture growth. These strains were cultured in 500 mL of L1 medium. Sampling was performed as described above at days 7, 11, and 13 post inoculation for strains 119-A4 and 119-B3 and at days 4, 6, and 11 and 4, 5, 6, 7, 11, and 13 post inoculation for strains MS2 and MS3, respectively.

### 4.3. Direct Competitive ELISA 

The main method used to test most of the cultures examined was the competitive ELISA assay for the detection of domoic acid (Eurofins Abraxis, Warminster, MA, USA). The cultures were processed according to the manufacturer’s guidelines. A constant temperature was maintained during plate preparation and pipetting. The reliability of the procedure was verified using internal and external controls and standards. The absorbance reader was turned on for an extended period of time prior to measurement to allow the light source to settle. This method is approved by the European Commission as a screening method for the determination of DA in shellfish (Commission Implementing Regulation (EU) 2019/627). Samples were diluted 1:25 with the dilution buffer provided and further diluted to 1:50 if the signal was still saturated. If the signal was borderline or unquantifiable, the samples were diluted less to 1:10. Some samples were not reanalyzed at higher or lower dilutions and are only reported as positive. Due to inconsistencies between ELISA and the analytical methods, some samples were measured multiple times by ELISA to confirm the presence or absence of toxin.

### 4.4. HPLC-UV Method for Selected Cultures

This method is generally intended for regulatory purposes, as it is sufficiently sensitive to detect toxicity when the toxin concentration approaches or exceeds the threshold. It is also capable of accurately measuring high concentrations of DA in cultures. Domoic acid content was determined after chromatographic separation on a reversed phase column (C18 reversed phase, 5 µm, 250 mm × 4.6 mm) under isocratic conditions (acetonitrile 5% with TFA 0.1% *v*/*v*). The analysis was performed with a UV-VIS detector set to 242 nm.

The amount of domoic acid was calculated using a certified DA standard from the National Research Council of Canada, which was used to prepare three dilutions that were used to calibrate the HPLC system.

### 4.5. LC-MS/MS for Selected Cultures

The LC-MS/MS analysis of domoic acid in extracts was performed using an Agilent UHPLC Infinity II (Agilent Technologies Inc., Santa Clara, CA, USA) equipped with an Agilent POROSHELL 120 EC C18, 2.1 × 100-mm 2.7-µm—LC column (Agilent Technologies Inc., Santa Clara, CA, USA) coupled to an Agilent 6460 Triple Quad Mass Spectrometer (Agilent Technologies Inc., Santa Clara, CA, USA). A certified DA standard from NRC-Canada was used to prepare five standard solutions that were used to calibrate the LC-MS/MS system in the multiple reaction monitoring (MRM) mode. The identification of the analyte was based on monitoring two ion products of DA (*m/z* 312 > 266, 312 > 161 from DA precursor ion (M + H) + *m/z* 312) in positive electrospray ionization (ESI+) mode. The most abundant fragment, 266, was selected for quantification, while the 161 ion was used for qualitative confirmation. A methanol/water solution of ammonium acetate/acetic acid was chosen the mobile phase for chromatographic separation. A total of 5 µL of the sample was injected into the LC-MS/MS system, and a 14 min gradient elution was used to separate the toxins.

### 4.6. ITS-2 Phylogeny Reconstruction with Tested Strains

The complete ITS region was sequenced as described in [13]. Sequences used for phylogenetic tree reconstruction were selected based on two factors: whether the publications under which they were published contained toxicity data and whether they were geographically representative. Phylogenetic trees based on the ITS2 region were constructed separately for each species with the phangron [50] and ape [51] packages implemented in R [52] using the implemented maximum likelihood algorithm with nearest neighbor interchange (NNI) optimization and a transversion model with estimated invariant sites and a gamma distribution of rates (TVM + G + I) that was established by the modelTest() command. Ten thousand bootstraps of the tree space were performed using the bootstrap.pml() command.

### 4.7. Amplification and Sequence Analysis of the dabA Gene

For the amplification of the *dabA* gene, we first unsuccessfully tried the primers published in [9]. Then, we designed new primers (Table 2) based on the alignment of sequences from shotgun sequencing of the *P. multistriata* genome [26], BioProject accession: PRJEB9419 (https://www.ncbi.nlm.nih.gov/bioproject/PRJEB9419/; accessed on 13 April 2021) and [9]. Internal sequencing primers were also designed to complete the gene region. Amplification was performed using Phusion HiFi Polymerase (New England Biolabs, Ipswich, MA, USA). For PCR reactions that resulted in nonspecific products, bands containing products of the appropriate size (~1500 bp) were excised from the agarose gels. To do this, products were run at 200 V for 30 s in pre-cut wells in the gel, followed by precipitation with 3M sodium acetate and isopropanol at −20 °C for one hour. The precipitates were then washed with 70% ethanol and dissolved in 1× Tris Low-EDTA (TLE). Products were also purified with exonuclease I and Alkaline Phosphatase—FastAP (Thermo Fisher Scientific, Waltham, MA, USA). Alternatively, bands were excised from the gel with a sterile scalpel when possible, and gel purification was performed with NucleoSpin Gel and PCR Clean-up (Machery-Nagel, Düren, Germany). The 3D structure of the predicted proteins was predicted using Phyre2 (http://www.sbg.bio.ic.ac.uk/phyre2/html/page.cgi?id=index; accessed on 22 January 2021) [53].

## Figures and Tables

**Table 1:** Summary of strains and tests performed on each strain and the concentrations of DA associated with them.

Species	Strain	Sampling Frequency ^1^	Partition Tested	Test Method ^2^	Result ^3^	DA Concentration^4^	ELISA Retest ^5^
*P. delicatissima*	219 A1	P	pDA	E	*-*		
	219 A2	P	pDA	E	*-*		
	219 A3	P	pDA	E	*-*		
	219 B1	P	pDA	E	*-*		
	219 B2	P	pDA	E	*-*		
	219 B3	P	pDA	E	*-*		
	219 B4	P	pDA	E	*-*		
	119 A2	P	pDA	E	*-*		
	119 A3	P	pDA	E	*-*		
	119 B3	P & C	pDA and dDA	E&H	+ (E)	1.5 fg/cell	<quant
	119 C1	P	pDA and dDA	E&H	+ (E)	NA	<quant
	119 C4	P	pDA	E	*-*		
*P. multistriata*	119 A4	P & C	pDA and dDA	E&H	+ (E&H)	pDA: 16–114 fg/cell; dDA: 6–257 ng/mL	
	119 C3	P	pDA and dDA	E&H	+ (E&H)	pDA: 121–160 fg/cell; dDA: 30–80	
	MS2	P & C	pDA and dDA	E; H; L	+ (E)	pDA: 0–32.8 fg/cell; dDA: 20 ng/mL	<quant
	MS3	P & C	pDA and dDA	E; H; L	+ (E)	pDA: 1.42–20.7 fg/cell; dDA: 14.6–38.5 ng/mL	<quant
	PN0DB2131216-A	P	pDA	L	-		
	PN0DB2131216-B	P	pDA	L	-		
	PN0DB2131216-C	P	pDA	L	+	0.217 fg/cell	
*P. galaxiae*—large	MA-919-C1	P	pDA and dDA	E&H	*-*		
	MA-919-A2	P	pDA and dDA	E&H	*-*		
	F919-C2L	P	pDA and dDA	E&H	*-*		
*P. galaxiae*— medium	F919-B1M	P	pDA and dDA	E&H	*-*		
*P. galaxiae*—bat-like	BAT2	P	pDA and dDA	E&H	o (E)	dDA: 13 ng/mL	<quant
*P. galaxiae*—small	B3S	P	pDA and dDA	E&H	+ (E)	dDA: 5–24.8 ng/mL; pDA > quant	>quant
	B2S	P	pDA and dDA	E&H	o (E)	dDA: 12.6 ng/mL	<quant
*P. mannii*	BF-819-B2	P	pDA and dDA	E&H	*-*		
	BF-819-A4	P	pDA and dDA	E&H	*-*		
	BD-919-A3	P	pDA and dDA	E&H	*-*		
	PNF_1020_5	P	pDA and dDA	E	o	<quant	
*P. subfraudulenta*	PNF_1020_1	P	pDA and dDA	E	*-*		
*P. calliantha*	PNF_1020_2	P	pDA and dDA	E	o	<quant	
	PN00BF281016-2A	P	pDA	L	-		

^1^ P, point; C, continuous. ^2^ E, ELISA; H, HPLC-UV; L, LC-MS/MS. ^3^ o indicates an inconclusive result. ^4^ NA, not available; the toxicity range is obtained from different tests of the same culture. ^5^ </> quant, below/above quantification.

**Table 2:** Primers used for amplification and sequencing of the *dabA* gene in *P. multistriata*.

Primer Name	Primer-Sequence	Type
DabA_multF	ATGAAATTTGCAACGTCCATTGTC	PCR
DabA_degF	ATGAARTTTGCAACRTCCATYGTC	PCR
N1_R	TCCAAAAACGCTTTCATCAA	PCR
N2_R	AACGCTTTCATCAATGGTTTGTGG	PCR
Internal_multistriataF	CGATTGGATGAAGATCCCTTCA	Sequencing
Internal_multistriataR	GCAGAAGTCGACCATCCA	Sequencing

## Data Availability

Sequence data is available in GenBank. Genomic resources are deposited at NIB MBP. Toxin analysis data are available from NIB MBP.

## Supplementary Materials

The following supporting information can be downloaded at: https://www.mdpi.com/article/10.3390/toxins14010060/s1, Figure S1. Homology modelling of the translated sequence of the *dabA* gene from *Pseudo-nitzschia multistriata*, strain MS3. Most of the secondary structures are recovered and the protein resembles the published crystalline structure. Table S1. ITS and *dabA* accession numbers of strains used in the phylogeny reconstruction.
