# Supplementary material for: Toxicity of the Diatom Genus Pseudo-nitzschia (Bacillariophyceae): Insights from Toxicity Tests and Genetic Screening in the Northern Adriatic Sea
Source: Toxins (Basel). 2022 Jan 15;14(1):60. doi: 10.3390/toxins14010060 (PMC8781606; doi:10.3390/toxins14010060)
Supplement: Supplementary file 1 [file toxins-14-00060-s001.zip › toxins-1531977-supplementary.pdf]

toxins

Supplementary

Toxicity of the Diatom Genus *Pseudo-nitzschia* (Bacillariophyceae): Insights from Toxicity Tests and Genetic Screening in the Northern Adriatic Sea

Timotej Turk Dermastia [1,2,*], Sonia Dall'Ara [3], Jožica Dolenc [4] and Patricija Mozetič [1]

1 Marine Biology Station Piran, National Institute of Biology, 6330 Piran, Slovenia; patricija.mozetic@nib.si
2 International Postgraduate School Jožef Stefan, 1000 Ljubljana, Slovenia
3 National Reference Laboratory for Marine Biotoxins, Centro Ricerche Marine, 47042 Cesenatico, Italy; sonia.dallara@centroricerchemarine.it
4 Institute of Food Safety, Feed and Environment, Veterinary Faculty, University of Ljubljana, 1000 Ljubljana, Slovenia; jozica.dolenc@vf.uni-lj.si
* Correspondence: timotej.turkdermastia@nib.si

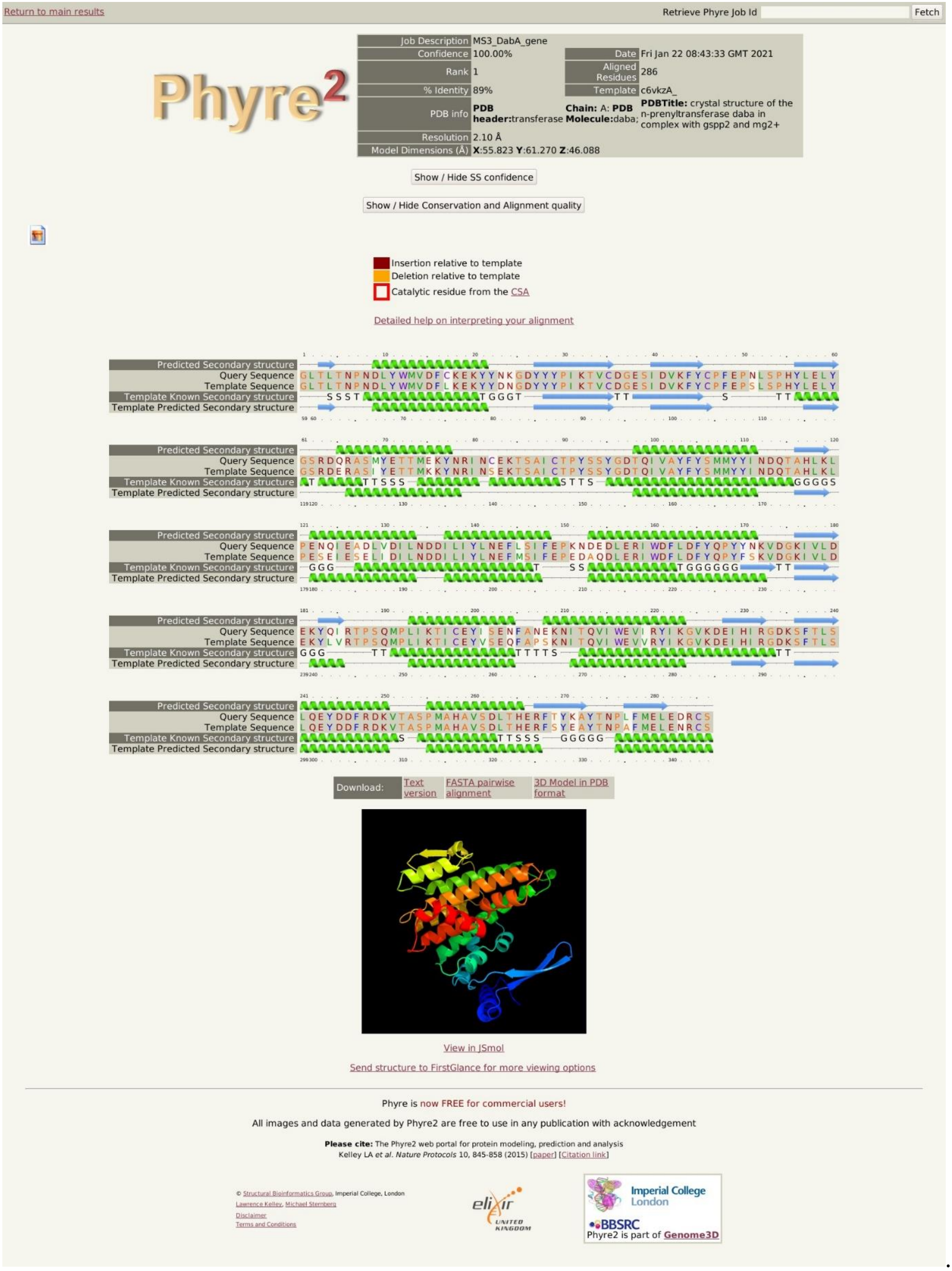

Figure S1. Homology modelling of the translated sequence of the *dabA* gene from *Pseudo-nitzschia multistriata*, strain MS3. Most of the secondary structures are recovered and the protein resembles the published crystalline structure.

Table S1. ITS accession numbers of strains used in the phylogeny reconstruction.

Species	Strain	Geographical Area	Genbank ITS Accession	GenBank *dabA* Accession	Reference for Toxicity
P. abrensis	PnMi81	Malaysia (Borneo)	KX572953		[1]
P. americana	PH25	E Australia (NSW)	KC017472		[2]
P. arctica	P2F2	Greenland	KT589421		[3]
P. arenysensis	CHB	E Australia (NSW)	KC017466		[4]
P. australis	Delta 2	Portugal (Aveiro)	EU684233		[5]
	L3.16	Namibia	MH376354		[6]
P. batesiana	PnMi32	Malaysia (Borneo)	KX572953		[1]
P. bipertita	MC4571	Malaysia (Borneo)	MT023649		[1]
P. bucculenta	L1.3	Namibia	MH376341		[6]
P. caciantha	PnSL05	E Australia (NSW)	KF482056.1		[7]
	COOG100215-1	E Australia (NSW)	LC154962		[7]
P. calliantha	(09)2A12	Greece (Aegean)	JF714901		[8]
	ICMB−158	Spain (Catalonia)	EU327378		[9]
	WAG	E Australia (NSW)	KC017463		[4]
	PNF 1020 2	NE Adriatic			This study
	PNF 1020 5	NE Adriatic			This study
	PNF 1020 3	NE Adriatic			This study
	BF−818−B2	NE Adriatic			This study
P. circumpora	PnSb58	Malaysia (Borneo)	JN252430		[10]
P. chiniana	MC3012	Taiwan Strait	MK411964		[11]
P. cuspidiata	MER	E Australia (NSW)	KC017465		[4]
P. cuspidiata var. *manzanillensis*	SQS−2017a	Mexico (Pacific)	KU527659		[12]
P. decipiens	S1.6	Namibia	MH376345		[6]
P. delicatissima	219 A1	NE Adriatic	MN634971.1		This study
	219 A2	NE Adriatic	MN634973.1		This study
	219 A3	NE Adriatic	MN634969.1		This study
	219 B1	NE Adriatic	MN634972.1		This study
	219 B2	NE Adriatic	MN634970.1		This study
	219 B3	NE Adriatic	MN634967.1		This study
	119 A2	NE Adriatic	MN634965.1		This study
	119 B3	NE Adriatic	MN634964.1		This study
	119 C1	NE Adriatic	MN634966.1		This study
	119 C4	NE Adriatic	MN634968.1		This study
	ICMB−102	Spain (Catalonia)	EU478793		[9]
	(10)4A5	Greece (Aegean)	JF714910.		[8]
	Pn100−07A2	Northwestern Atlantic Ocean (Gulf of Maine)	KF006826		[13]
P. dolorosa	S3.2	Namibia	MH376344		[6]
P. fraudulenta	S2.1	Namibia	MH376349		[6]
P. fryxelliana	NWFSC 241	E Pacific (Washington, USA)	JN050288		[14]
P. fukuyoi	PnLk02	Malaysia (Borneo)	JN252420		[15]

P. galaxiae	00MA090919-C1	NE Adriatic	MW527383		This study
	00MA090919-A2	NE Adriatic	MW527387		This study
	BAT2	NE Adriatic	MW527386		This study
	B1M	NE Adriatic	MW527388		This study
	B2S	NE Adriatic	MW527390		This study
	B3S	NE Adriatic	MW527391		This study
	C2L	NE Adriatic	MW527384		This study
	MC994	Taiwan Strait	MK411977		[11]
	(10)4A3	Greece (Aegean)	JF714915		[8]
P. hallegraeffii	CTD44 2	E Australia (NSW)	MF044023		[16]
P. hasleana	NWFSC 186	E Pacific (Washington, USA)	N050282		[14]
P. kodamae	Pnmi92	Malaysia (Borneo)	KR021310		[1]
	PnPd31	Malaysia (Malacca)	KF482051		[15]
P. limii	Pnmi16	Malaysia (Borneo)	KR021311		[1]
P. linea	ICMB−156	Spain (Catalonia)	FJ489632		[9]
P. lineola	NWFSC 188	Malaysia (Malacca)	MT040024		[15]
P. lundholmiae	Pnmi01	Malaysia (Borneo)	KR021315		[1]
P. mannii	(08)10A2	Greece (Aegean)	JF714905		[8]
P. micropora	PS90/1	E Australia (NSW)	KC017448		[2]
P. multiseries	COOG	E Australia (NSW)	KC017469		[4]
	Pn-1	Northwestern Atlantic Ocean (Chesapeake Bay))	DQ445651		[17]
P. multistriata	MS2	NE Adriatic	MW527382	MZ486437	This study
	MS3	NE Adriatic	MW527381	MZ486439	This study
	119 A3	NE Adriatic	MN634976.1		This study
	119 A4	NE Adriatic	MN634975.1	MZ486438	This study
	119 C4	NE Adriatic	MN634974.1	MZ486440	This study
	PH25C	E Australia (NSW)	KC017470		[4]
	MC4171	Taiwan Strait	MK411975		[2]
	Pnmi07	Malaysia (Borneo)	KR021314		[1]
P. nanaoensis	MC4213	South China Sea (Guangdong coast)	MG787882		[18]
P. plurisecta	L3.9	Namibia	MH376350		[6]
P. pseudodelicatis-sima	P−11	Portugal	AY257854.		[19]
	(09)10A4	Greece (Aegean)	JF714921		[8]
P. pungens	ICMB−143	Spain (Catalonia)	EU327366		[9]
	(10)4A2	Greece (Aegean)	JF714922		[8]
P. pungens var. *cingulata*	L3.13	Namibia	MH376355		[6]
P. pungens var. *aveirensis*	Alfa 1	Portugal (Aveiro)	EU684234		[5]
P. qiana	MC3033	Taiwan Strait	MK412849		[2]
P. sabit	MC4399	South China Sea (Guangdong coast)	MT040005		[20]

P. seriata	Pn114−08C2	Northwestern Atlantic Ocean (Gulf of Maine)	KF006824	[13]
P. simulans	MC940	South China Sea (Guangdong coast)	MF374771	[21]
	MC984	South China Sea (Guangdong coast)	MF374772	[21]
P. subcurvata	M11-04	Antarctica (Queen Maud Land)	MT420907	[22]
	M5-3	Antarctica (Queen Maud Land)	MT420903	[22]
P. subfraudulenta	PnMi155	Malaysia (Borneo)	KR021296.	[1]
	(08)8A3	Greece (Aegean)	JF714929	[8]
P. subpacifica	MC4435	South China Sea (Guangdong coast)	MT040017	[20]
P. uniseriata	MC4196	South China Sea (Guangdong coast)	MT040024	[20]
P. yuensis	MC3209	South China Sea (Guangdong coast)	MT040025	[20]

References

1. Teng, S.T.; Tan, S.N.; Lim, H.C.; Dao, V.H.; Bates, S.S.; Leaw, C.P. High diversity of *Pseudo-nitzschia* along the northern coast of Sarawak (Malaysian Borneo), with descriptions of *P. bipertita* sp. nov. and *P. limii* sp. nov. (Bacillariophyceae). *J. Phycol.* **2016**, *52*, 973–989, doi:10.1111/jpy.12448.
2. Ajani, P.A.; Murray, S.; Hallegraeff, G.; Brett, S.; Armand, L. First reports of *Pseudo-nitzschia micropora* and *P. hasleana* (Bacillariaceae) from the Southern Hemisphere: Morphological, molecular and toxicological characterization. *Phycol. Res.* **2013**, *61*, 237–248, doi:10.1111/pre.12020.
3. Lundholm, N.; Krock, B.; John, U.; Skov, J.; Cheng, J.; Pančić, M.; Wohlrab, S.; Rigby, K.; Nielsen, T.G.; Selander, E.; et al. Induction of domoic acid production in diatoms—Types of grazers and diatoms are important. *Harmful Algae* **2018**, *79*, 64–73, doi:10.1016/j.hal.2018.06.005.
4. Ajani, P.A.; Murray, S.; Hallegraeff, G.; Lundholm, N.; Gillings, M.; Brett, S.; Armand, L. The diatom genus *Pseudo-nitzschia* (Bacillariophyceae) in New South Wales, Australia: Morphotaxonomy, molecular phylogeny, toxicity, and distribution. *J. Phycol.* **2013**, *49*, 765–785, doi:10.1111/jpy.12087.
5. Churro, C.I.; Carreira, C.C.; Rodrigues, F.J.; Craveiro, S.C.; Calado, A.J.; Casteleyn, G.; Lundholm, N. Diversity and abundance of potentially toxic *Pseudo-nitzschia* Peragallo in Aveiro coastal lagoon, Portugal and description of a new variety, *P. pungens* var. *aveirensis* var. nov. *Diatom Res.* **2009**, *24*, 35–62, doi:10.1080/0269249X.2009.9705782.
6. Gai, F.F.; Hedemand, C.K.; Louw, D.C.; Grobler, K.; Krock, B.; Moestrup, Ø.; Lundholm, N. Morphological, molecular and toxigenic characteristics of Namibian *Pseudo-nitzschia* species – including *Pseudo-nitzschia bucculenta* sp. nov. *Harmful Algae* **2018**, *76*, 80–95, doi:10.1016/j.hal.2018.05.003.
7. Ajani, P.; Kim, J.H.; Han, M.S.; Murray, S.A. The first report of the potentially harmful diatom *Pseudo-nitzschia caciantha* from Australian coastal waters. *Phycol. Res.* **2016**, *64*, 312–317, doi:10.1111/pre.12142.
8. Moschandreou, K.K.; Baxevanis, A.D.; Katikou, P.; Papaefthimiou, D.; Nikolaidis, G.; Abatzopoulos, T.J. Inter- and intra-specific diversity of *Pseudo-nitzschia* (Bacillariophyceae) in the northeastern Mediterranean. *Eur. J. Phycol.* **2012**, *47*, 321–339, doi:10.1080/09670262.2012.713998.
9. Quijano-Scheggia, S.; Garcés, E.; Andree, K.B.; de la Iglesia, P.; Diogène, J.; Fortuño, J.M.; Camp, J. Especies *Pseudo-nitzschia* en la costa Catalana: Caracterización y contribución al conocimiento actual de la distribución del género en el mar Mediterráneo. *Sci. Mar.* **2010**, *74*, 395–410, doi:10.3989/scimar.2010.74n2395.
10. Lim, H.C.; Leaw, C.P.; Su, S.N.P.; Teng, S.T.; Usup, G.; Mohammad-Noor, N.; Lundholm, N.; Kotaki, Y.; Lim, P.T. Morphology and molecular characterization of *Pseudo-nitzschia* (Bacillariophyceae) from Malaysian Borneo, including the new species *Pseudo-nitzschia circumpora* sp. nov. *J. Phycol.* **2012**, *48*, 1232–1247, doi:10.1111/j.1529-8817.2012.01213.x.
11. Huang, C.X.; Dong, H.C.; Lundholm, N.; Teng, S.T.; Zheng, G.C.; Tan, Z.J.; Lim, P.T.; Li, Y. Species composition and toxicity of the genus *Pseudo-nitzschia* in Taiwan Strait, including *P. chiniana* sp. nov. and *P. qiana* sp. nov. *Harmful Algae* **2019**, *84*, 195–209, doi:10.1016/j.hal.2019.04.003.
12. Rivera-Vilarelle, M.; Valdez-Velázquez, L.L.; Quijano-Scheggia, S.I. Description of *Pseudo-nitzschia cuspidata* var. *manzanillensis* var. nov. (Bacillariophyceae): morphology and molecular characterization of a variety from the central Mexican pacific. *Diatom Res.* **2018**, *33*, 55–68, doi:10.1080/0269249X.2018.1426633.

13. Fernandes, L.F.; Hubbard, K.A.; Richlen, M.L.; Smith, J.; Bates, S.S.; Ehrman, J.; Léger, C.; Mafra, L.L.; Kulis, D.; Quilliam, M.; et al. Diversity and toxicity of the diatom *Pseudo-nitzschia* Peragallo in the Gulf of Maine, Northwestern Atlantic Ocean. *Deep. Res. Part II Top. Stud. Oceanogr.* **2014**, *103*, 139–162, doi:10.1016/j.dsr2.2013.06.022.
14. Lundholm, N.; Bates, S.S.; Baugh, K.A.; Bill, B.D.; Connell, L.B.; Léger, C.; Trainer, V.L. Cryptic and pseudo-cryptic diversity in diatoms-with descriptions of *Pseudo-nitzschia hasleana* sp. nov. and *P. fryxelliana* sp. nov. *J. Phycol.* **2012**, *48*, 436–454, doi:10.1111/j.1529-8817.2012.01132.x.
15. Teng, S.T.; Lim, H.C.; Lim, P.T.; Dao, V.H.; Bates, S.S.; Leaw, C.P. *Pseudo-nitzschia kodamae* sp. nov. (Bacillariophyceae), a toxigenic species from the strait of Malacca, Malaysia. *Harmful Algae* **2014**, *34*, 17–28, doi:10.1016/j.hal.2014.02.005.
16. Ajani, P.A.; Verma, A.; Lassudrie, M.; Doblin, M.A.; Murray, S.A. A new diatom species P. *hallegraeffii* sp. nov. belonging to the toxic genus *Pseudo-nitzschia* (Bacillariophyceae) from the East Australian Current. *PLoS One* **2018**, *13*, e0195622, doi:10.1371/journal.pone.0195622.
17. Thessen, A.E.; Bowers, H.A.; Stoecker, D.K. Intra- and interspecies differences in growth and toxicity of *Pseudo-nitzschia* while using different nitrogen sources. *Harmful Algae* **2009**, *8*, 792–810, doi:10.1016/j.hal.2009.01.003.
18. Li, Y.; Dong, H.C.; Teng, S.T.; Bates, S.S.; Lim, P.T. *Pseudo-nitzschia nanaoensis* sp. nov. (Bacillariophyceae) from the Chinese coast of the South China Sea. *J. Phycol.* **2018**, *54*, 918–922, doi:10.1111/jpy.12791.
19. Lundholm, N.; Moestrup, Ø.; Hasle, G.R.; Hoef-Emden, K. A study of the *Pseudo-nitzschia pseudodelicatissima/cuspidata* complex (Bacillariophyceae): What is *P. pseudodelicatissima*? *J. Phycol.* **2003**, *39*, 797–813, doi:10.1046/j.1529-8817.2003.02031.x.
20. Dong, H.C.; Lundholm, N.; Teng, S.T.; Li, A.; Wang, C.; Hu, Y.; Li, Y. Occurrence of *Pseudo-nitzschia* species and associated domoic acid production along the Guangdong coast, South China Sea. *Harmful Algae* **2020**, *98*, 101899, doi:10.1016/j.hal.2020.101899.
21. Li, Y.; Huang, C.X.; Xu, G.S.; Lundholm, N.; Teng, S.T.; Wu, H.; Tan, Z. *Pseudo-nitzschia simulans* sp. nov. (Bacillariophyceae), the first domoic acid producer from Chinese waters. *Harmful Algae* **2017**, *67*, 119–130, doi:10.1016/j.hal.2017.06.008.
22. Olesen, A.J.; Leithoff, A.; Altenburger, A.; Krock, B.; Beszteri, B.; Eggers, S.L.; Lundholm, N. First Evidence of the Toxin Domoic Acid in Antarctic Diatom Species. *Toxins (Basel).* **2021**, *13*, 1–11, doi:10.3390/toxins13020093.
